# Crystal structures of 4-phenyl­piperazin-1-ium 6-chloro-5-ethyl-2,4-dioxopyrimidin-1-ide and 4-phenyl­piperazin-1-ium 6-chloro-5-isopropyl-2,4-dioxopyrimidin-1-ide

**DOI:** 10.1107/S2056989015013298

**Published:** 2015-07-22

**Authors:** Monirah A. Al-Alshaikh, Ali A. El-Emam, Omar A. Al-Deeb, Mohammed S. M. Abdelbaky, Santiago Garcia-Granda

**Affiliations:** a Department of Chemistry, College of Sciences, King Saud University, Riyadh 11451, Saudi Arabia; b Department of Pharmaceutical Chemistry, College of Pharmacy, King Saud University, Riyadh 11451, Saudi Arabia; c Department of Physical and Analytical Chemistry, Faculty of Chemistry, Oviedo University-CINN, Oviedo 33006, Spain

**Keywords:** crystal structure, piperazinium salts, anti­cancer activity, hydrogen bonding

## Abstract

The title mol­ecular salts, C_10_H_15_N_2_^+^·C_6_H_6_ClN_2_O_2_^−^, (I), and C_10_H_15_N_2_^+^·C_7_H_8_ClN_2_O_2_^−^, (II), consist of 4-phenyl­piperazin-1-ium cations with a 6-chloro-5-ethyl-2,4-dioxopyrimidin-1-ide anion in (I) and a 6-chloro-5-isopropyl-2,4-dioxopyrimidin-1-ide anion in (II). Salt (I) crystallizes with two independent cations and anions in the asymmetric unit. In the crystal structures of both salts, the ions are linked *via* N—H⋯O and N—H⋯N hydrogen bonds, forming sheets which are parallel to (100) in (I) and to (001) in (II). In (I), the sheets are linked *via* C—H⋯Cl hydrogen bonds, forming a three-dimensional framework.

## Abstract 2

In the crystals of the two novel piperazinium salts with a 6-chloro-5-ethyl-2,4-dioxopyrimidin-1-ide anion in (I) and a 6-chloro-5-isopropyl-2,4-dioxopyrimidin-1-ide anion in (II), the anions and cations are linked *via* N—H⋯O and N—H⋯N hydrogen bonds, forming sheets which are parallel to (100) in (I) and to (001) in (II). Salt (I) crystallizes with two independent 6-chloro-5-ethyl­uracil anions and two 1-phenyl­piperazine cations in the asymmetric unit.

## Chemical context   

2,4-Dioxo­pyrimidine derivatives (uracils) and their related analogues are known for their diverse chemotherapeutic activities including anti­cancer activity (Ghoshal & Jacob, 1997 ▸; Spáčilová *et al.*, 2007 ▸; Blokhina *et al.*, 1972 ▸), anti-HIV activity (Tanaka *et al.*, 1995 ▸; El-Emam *et al.*, 2004 ▸) and anti­bacterial activity (Al-Turkistani *et al.*, 2011 ▸). In addition, the piperazine nucleus constitutes the core pharmacophore of several biologically active compounds which display anti­viral (Romero *et al.*, 1994 ▸, 1996 ▸), anti­cancer (Fytas *et al.*, 2015 ▸; Kamal *et al.*, 2015 ▸; Arnatt *et al.*, 2014 ▸), anti­tubercular and anti­bacterial (Nagesh *et al.*, 2014 ▸; Peng *et al.*, 2015 ▸; Kapić *et al.*, 2011 ▸; Wang *et al.*, 2014 ▸) and central nervous system activities (Bender *et al.*, 2014 ▸; Bali *et al.*, 2010 ▸). 
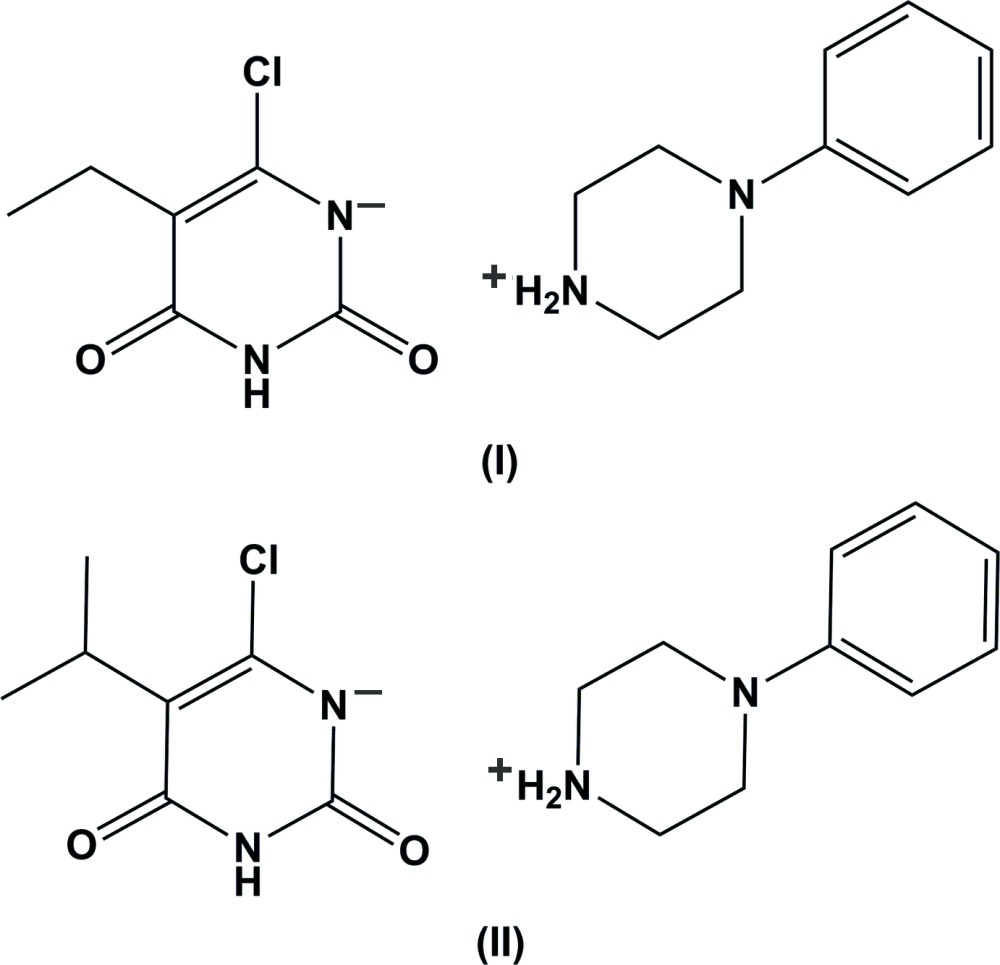


As a result of the relative acidity of 2,4-dioxo­pyrimidines (Kurinovich & Lee, 2002 ▸; Jang *et al.*, 2001 ▸; Nguyen *et al.*, 1998 ▸), the title piperazinium salts were isolated as minor byproducts during the reaction of 1-phenyl­piperazine with 5-alkyl-6-chloro­uracils (Al-Turkistani *et al.*, 2011 ▸). In a continuation of our inter­est in the structures of piperazinium salts (Al-Omary *et al.*, 2014 ▸), we report herein on the isolation and crystal structures of these two new piperazinium salts, (I) and (II).

## Structural commentary   

**Figure 1:**
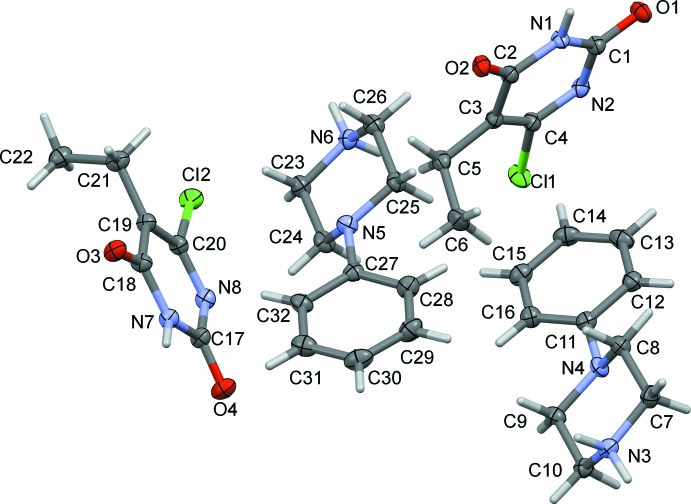
The mol­ecular structure of compound (I), showing the atom labelling. Displacement ellipsoids are drawn at the 50% probability level.

The mol­ecular structures of the title salts (I) and (II) are illustrated in Figs. 1 ▸ and 2 ▸, respectively. Compound (I) crystallizes with two independent 4-phenyl­piperazin-1-ium cations (*A* and *B*) and two independent 6-chloro-5-ethyl-2, 4-dioxopyrimidin-1-ide anions (*C* and *D*) in the asymmetric unit. In both compounds, the piperazine rings adopt a distorted chair conformation with a positively charged protonated N atom. In compound (I), the mean plane of the piperazine ring makes a dihedral angle of 34.8 (2)° with the attached phenyl ring in cation *A*, and 39.7 (2)° in cation *B*. The equivalent dihedral angle is 39.61 (9)° in the cation of compound (II). In the uracil anions, the pyrimidine rings are almost planar with r.m.s. deviations of 0.008 Å in both anions (*C* and *D*) of compound (I), and 0.024 Å in compound (II).

## Supra­molecular features   

**Figure 3:**
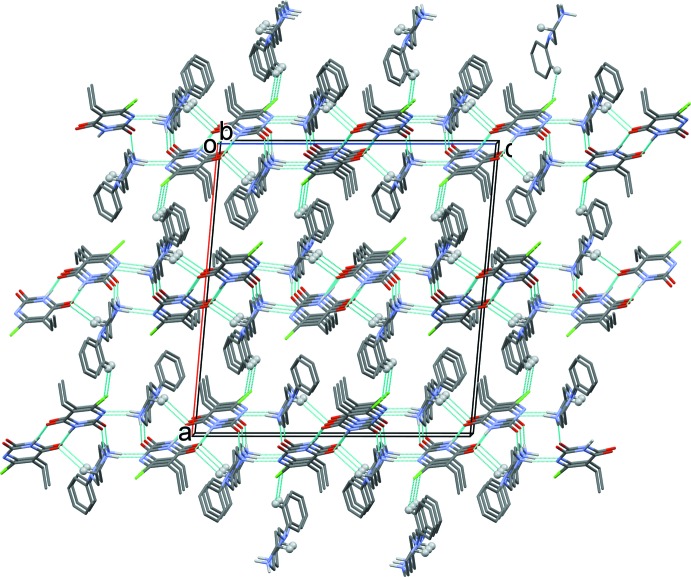
The crystal packing of compound (I), viewed along the *b* axis, showing the most relevant hydrogen bonding (dashed lines; see Table 1 ▸).

In the crystal of (I), two tetra­nuclear units are formed, involving cation *A* and anion *C*, and cation *B* and anion *D*, *via* N—H⋯O and C—H⋯O hydrogen bonds. These units are linked *via* N—H⋯N hydrogen bonds, forming separate *A*/*B* and *C*/*D* sheets parallel to the *bc* plane (Table 1 ▸ and Fig. 3 ▸). The sheets are linked *via* C—H⋯Cl hydrogen bonds, forming a three-dimensional framework (Fig. 3 ▸ and Table 1 ▸).

**Figure 4:**
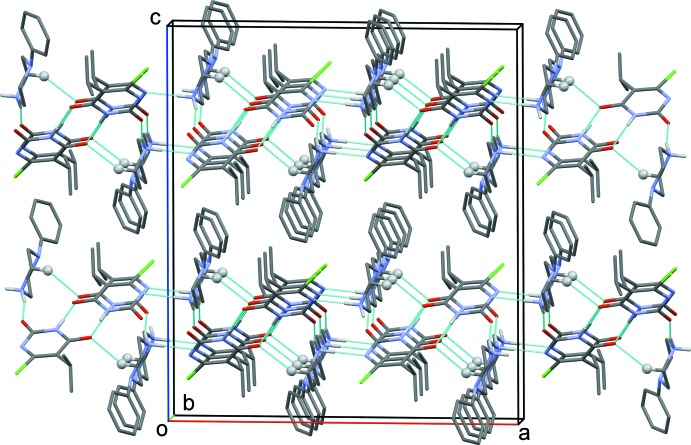
The crystal packing of compound (II), viewed along the *b* axis, showing the most relevant hydrogen bonding (dashed lines; see Table 2 ▸).

In the crystal of (II), the cation and anion are linked by N—H⋯O and C—H⋯O hydrogen bonds, forming chains extending along the *b-*axis direction. The chains are linked *via* N—H⋯N hydrogen bonds, forming sheets lying parallel to the *ac* plane (Table 2 ▸ and Fig. 4 ▸).

## Database survey   

A search of the Cambridge Structural Database (Version 5.36, last update November 2014; Groom & Allen, 2014 ▸) for the anion 6-chloro-5-ethyl-2,4-dioxopyrimidin-1-ide, present in compound (I), gave no hits, while for the anion 6-chloro-5-isopropyl-2,4-dioxopyrimidin-1-ide, present in compound (II), one hit was obtained, with the cation 4-(2-meth­oxy­phen­yl)piperazin-1-ium (Al-Omary *et al.*, 2014 ▸).

## Synthesis and crystallization   

**Compound (I)**: A mixture of 6-chloro-5-ethyl­uracil (349 mg, 2.0 mmol), 1-phenyl­piperazine (325 mg, 2.0 mmol) and anhydrous potassium carbonate (276 mg, 2.0 mmol), in ethanol (8 ml), was heated under reflux for 6 h. On cooling, the precipitate, thus formed was separated by filtration to yield 306 mg (51%) of 5-ethyl-6-(4-phenyl-1-piperazin­yl)uracil. The filtrate was concentrated by vacuum distillation to 5 ml and allowed to stand at room temperature overnight to yield compound (I) as colourless crystals (m.p.: 459–461 K). ^1^H NMR (DMSO-*d*6, 500.13 MHz): δ 0.93 (*t*, 3H, CH_3_, *J* = 7.0 Hz), 2.35 (*q*, 2H, CH_2_), 3.25 (*s*, 4H, piperazine-H), 3.45 (*s*, 4H, piperazine-H), 6.83–6.95 (*m*, 3H, Ar—H), 7.21 (*d*, 2H, Ar—H, *J* = 6.6 Hz), 8.15–8.17 (*m*, 2H, NH_2_), 10.83 (*s*, 1H, NH). ^13^C NMR (DMSO-*d*6, 125.76 MHz): δ 13.80 (CH_3_), 19.55 (CH_2_), 44.18, 47.86 (piperazine-C), 116.32, 119.62, 128.44, 150.70 (Ar—C), 108.88, 153.90, 155.94, 164.80 (pyrimidine-C),

**Compound (II)**: 6-Chloro-5-iso­propyl­uracil (377 mg, 2.0 mmol), 1-phenyl­piperazine (325 mg, 2.0 mmol) and anhydrous potassium carbonate (276 mg, 2.0 mmol), in ethanol (8 ml), was heated under reflux for 6 h. On cooling, the precipitate thus formed was separated by filtration to yield 566 mg (90%) of 5-isopropyl-6-(4-phenyl-1-piperazin­yl)uracil. The filtrate was concentrated by vacuum distillation to 5 ml and allowed to stand at room temperature overnight to yield compound (II) as colourless crystals (m.p.: 473–475 K). ^1^H NMR (DMSO-*d*_6_, 500.13 MHz): δ 1.20 (*d*, 6H, CH_3_, *J* = 7.8 Hz), 2.52–2.56 (*m*, 1H, CH), 3.18 (*s*, 4H, piperazine-H), 3.24 (*s*, 4H, piperazine-H), 6.88–7.02 (*m*, 3H, Ar—H), 7.20–7.22 (*m*, 2H, Ar—H), 8.04–8.08 (*m*, 2H, NH_2_), 11.02 (*s*, 1H, NH). ^13^C NMR (DMSO-*d*_6_, 125.76 MHz): δ 19.98 (CH_3_), 27.0 (CH), 44.50, 47.98 (piperazine-C), 116.16, 119.80, 129.04, 150.0 (Ar—C), 110.82, 152.30, 154.04, 164.06 (pyrimidine-C).

## Refinement   

Crystal data, data collection and structure refinement details are summarized in Table 3 ▸. The H atoms were included in calculated positions and treated as riding atoms: N—H = 0.86–0.90 Å, C—H = 0.95–1.00 Å with *U*_iso_(H) = 1.5*U*_eq_(C) for methyl H atoms and 1.2*U*_eq_(N,C) for other H atoms.

## Supplementary Material

Crystal structure: contains datablock(s) I, II, Global. DOI: 10.1107/S2056989015013298/su5162sup1.cif

Structure factors: contains datablock(s) I. DOI: 10.1107/S2056989015013298/su5162Isup2.hkl

Structure factors: contains datablock(s) II. DOI: 10.1107/S2056989015013298/su5162IIsup3.hkl

Supporting information file. DOI: 10.1107/S2056989015013298/su5162Isup4.cml

Supporting information file. DOI: 10.1107/S2056989015013298/su5162IIsup5.cml

CCDC references: 1412124, 1412123

Additional supporting information:  crystallographic information; 3D view; checkCIF report

## Figures and Tables

**Figure 2:**
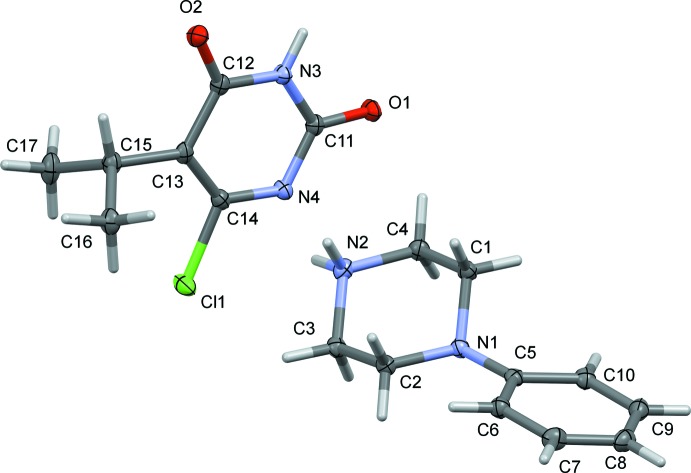
The mol­ecular structure of compound (II), showing the atom labelling. Displacement ellipsoids are drawn at the 50% probability level.

**Table 1:** Hydrogen-bond geometry (Å, °) for (I)

*D*—H⋯*A*	*D*—H	H⋯*A*	*D*⋯*A*	*D*—H⋯*A*
N1—H1⋯O2^i^	0.86	2.00	2.859 (4)	173
N3—H3*A*⋯O1^ii^	0.89	2.83	3.465 (4)	129
N6—H6*A*⋯O4^iii^	0.89	1.81	2.681 (5)	165
N7—H7⋯O3^iv^	0.86	2.02	2.873 (4)	174
N3—H3*A*⋯N2^ii^	0.89	1.92	2.808 (4)	174
N6—H6*B*⋯N8^v^	0.89	1.92	2.798 (5)	169
C10—H10*B*⋯O2^vi^	0.97	2.46	3.355 (5)	154
C26—H26*A*⋯O3^vii^	0.97	2.58	3.444 (5)	147
C16—H16⋯Cl2^viii^	0.93	2.80	3.462 (4)	129

Symmetry codes: (i) 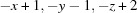; (ii) 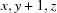; (iii) 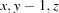; (iv) 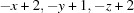; (v) 
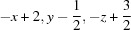
; (vi) 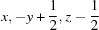; (vii) 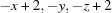; (viii) 
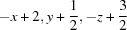
.

**Table 2:** Hydrogen-bond geometry (Å, °) for (II)

*D*—H⋯*A*	*D*—H	H⋯*A*	*D*⋯*A*	*D*—H⋯*A*
N2—H2*N*⋯N4	0.89	1.93	2.813 (2)	174
N2—H3*N*⋯O1^i^	0.89	1.84	2.705 (2)	164
N3—H3⋯O2^ii^	0.86	1.98	2.834 (2)	174
C3—H3*A*⋯O2^iii^	0.97	2.54	3.394 (2)	147

Symmetry codes: (i) 
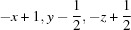
; (ii) 
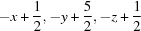
; (iii) 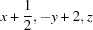.

**Table 3:** Experimental details

	(I)	(II)
Crystal data		
Chemical formula	C_10_H_15_N_2_^+^·C_6_H_6_ClN_2_O_2_^−^	C_10_H_15_N_2_^+^·C_7_H_8_ClN_2_O_2_^−^
*M*_r_	336.82	350.84
Crystal system, space group	Monoclinic, *P*2_1_/*c*	Monoclinic, *I*2/*a*
Temperature (K)	293	101
*a*, *b*, *c* (Å)	21.676 (1), 7.6446 (5), 20.5444 (8)	20.5012 (3), 7.4565 (1), 23.1414 (3)
β (°)	95.065 (5)	90.639 (1)
*V* (Å^3^)	3391.0 (3)	3537.34 (8)
*Z*	8	8
Radiation type	Cu *K*α	Cu *K*α
μ (mm^−1^)	2.12	2.05
Crystal size (mm)	0.17 × 0.08 × 0.06	0.34 × 0.13 × 0.09

Data collection		
Diffractometer	Agilent Xcalibur Ruby Gemini	Agilent Xcalibur Ruby Gemini
Absorption correction	Multi-scan (*CrysAlis PRO*; Agilent, 2014 ▸)	Multi-scan (*CrysAlis PRO*; Agilent, 2014 ▸)
*T*_min_, *T*_max_	0.809, 0.880	0.760, 0.828
No. of measured, independent and observed [*I* > 2σ(*I*)] reflections	32461, 6532, 3596	13174, 3396, 2926
*R*_int_	0.135	0.069
(sin θ/λ)_max_ (Å^−1^)	0.612	0.612

Refinement		
*R*[*F* ^2^ > 2σ(*F* ^2^)], *wR*(*F* ^2^), *S*	0.066, 0.185, 1.01	0.044, 0.122, 1.03
No. of reflections	6457	3346
No. of parameters	415	217
H-atom treatment	H-atom parameters constrained	H-atom parameters constrained
Δρ_max_, Δρ_min_ (e Å^−3^)	0.42, −0.36	0.55, −0.56

Computer programs: *CrysAlis PRO* (Agilent, 2014 ▸), *SIR2011* (Burla *et al.*, 2012 ▸), *Mercury* (Macrae *et al.*, 2008 ▸), *SHELXL2014* (Sheldrick, 2015 ▸), *PLATON* (Spek, 2009 ▸) and *publCIF* (Westrip, 2010 ▸).

## supplementary crystallographic information

### (I) 4-Phenylpiperazin-1-ium 6-chloro-5-ethyl-2,4-dioxopyrimidin-1-ide Crystal data

**Table:**

C_10_H_15_N_2_^+^·C_6_H_6_ClN_2_O_2_^−^	*F*(000) = 1424
*M**_r_* = 336.82	*D*_x_ = 1.319 Mg m^−^^3^
Monoclinic, *P*2_1_/*c*	Cu *K*α radiation, λ = 1.54184 Å
*a* = 21.676 (1) Å	Cell parameters from 2418 reflections
*b* = 7.6446 (5) Å	θ = 4.1–70.3°
*c* = 20.5444 (8) Å	µ = 2.12 mm^−^^1^
β = 95.065 (5)°	*T* = 293 K
*V* = 3391.0 (3) Å^3^	Prism, colourless
*Z* = 8	0.17 × 0.08 × 0.06 mm

### (I) 4-Phenylpiperazin-1-ium 6-chloro-5-ethyl-2,4-dioxopyrimidin-1-ide Data collection

**Table:**

Agilent Xcalibur Ruby Gemini diffractometer	6532 independent reflections
Radiation source: Enhance (Cu) X-ray Source	3596 reflections with *I* > 2σ(*I*)
Detector resolution: 10.2673 pixels mm^-1^	*R*_int_ = 0.135
ω scans	θ_max_ = 70.7°, θ_min_ = 4.1°
Absorption correction: multi-scan (CrysAlis PRO; Agilent, 2014)	*h* = −26→26
*T*_min_ = 0.809, *T*_max_ = 0.880	*k* = −9→9
32461 measured reflections	*l* = −21→25

### (I) 4-Phenylpiperazin-1-ium 6-chloro-5-ethyl-2,4-dioxopyrimidin-1-ide Refinement

**Table:**

Refinement on *F*^2^	Primary atom site location: structure-invariant direct methods
Least-squares matrix: full	Secondary atom site location: difference Fourier map
*R*[*F*^2^ > 2σ(*F*^2^)] = 0.066	Hydrogen site location: inferred from neighbouring sites
*wR*(*F*^2^) = 0.185	H-atom parameters constrained
*S* = 1.01	*w* = 1/[σ^2^(*F*_o_^2^) + (0.0563*P*)^2^] where *P* = (*F*_o_^2^ + 2*F*_c_^2^)/3
6457 reflections	(Δ/σ)_max_ = 0.001
415 parameters	Δρ_max_ = 0.42 e Å^−^^3^
0 restraints	Δρ_min_ = −0.36 e Å^−^^3^

### (I) 4-Phenylpiperazin-1-ium 6-chloro-5-ethyl-2,4-dioxopyrimidin-1-ide Special details

**Table:**

Geometry. All e.s.d.'s (except the e.s.d. in the dihedral angle between two l.s. planes) are estimated using the full covariance matrix. The cell e.s.d.'s are taken into account individually in the estimation of e.s.d.'s in distances, angles and torsion angles; correlations between e.s.d.'s in cell parameters are only used when they are defined by crystal symmetry. An approximate (isotropic) treatment of cell e.s.d.'s is used for estimating e.s.d.'s involving l.s. planes.

### (I) 4-Phenylpiperazin-1-ium 6-chloro-5-ethyl-2,4-dioxopyrimidin-1-ide Fractional atomic coordinates and isotropic or equivalent isotropic displacement parameters (Å^2^)

**Table:**

	*x*	*y*	*z*	*U*_iso_*/*U*_eq_	
Cl2	1.13235 (5)	0.04012 (14)	0.82294 (5)	0.0408 (3)	
Cl1	0.65035 (5)	−0.09582 (15)	0.82219 (5)	0.0437 (3)	
O1	0.51594 (14)	−0.6011 (4)	0.82514 (13)	0.0381 (7)	
O2	0.55114 (13)	−0.3192 (4)	1.02030 (13)	0.0344 (7)	
O3	1.04166 (14)	0.2995 (4)	1.02077 (13)	0.0378 (7)	
N1	0.53525 (15)	−0.4583 (4)	0.92238 (15)	0.0288 (8)	
H1	0.5119	−0.5322	0.9401	0.035*	
O4	1.02351 (14)	0.5927 (4)	0.82725 (14)	0.0415 (8)	
N7	1.03409 (15)	0.4421 (4)	0.92325 (15)	0.0306 (8)	
H7	1.0140	0.5236	0.9410	0.037*	
N2	0.57984 (15)	−0.3662 (4)	0.82841 (15)	0.0301 (8)	
N8	1.07502 (16)	0.3338 (5)	0.82954 (16)	0.0330 (8)	
N3	0.56236 (15)	0.6236 (4)	0.69138 (16)	0.0320 (8)	
H3A	0.5695	0.6194	0.7347	0.038*	
H3B	0.5338	0.7053	0.6817	0.038*	
N4	0.64335 (15)	0.3661 (5)	0.64533 (16)	0.0330 (8)	
N5	0.86014 (15)	0.0992 (4)	0.85634 (16)	0.0325 (8)	
N6	0.93121 (15)	−0.1748 (4)	0.80703 (16)	0.0337 (8)	
H6A	0.9565	−0.2655	0.8153	0.040*	
H6B	0.9245	−0.1637	0.7639	0.040*	
C1	0.54296 (19)	−0.4798 (5)	0.85679 (19)	0.0313 (9)	
C18	1.05419 (19)	0.3021 (5)	0.9626 (2)	0.0328 (10)	
C17	1.04364 (19)	0.4618 (6)	0.85811 (19)	0.0313 (9)	
C4	0.60638 (18)	−0.2374 (5)	0.8663 (2)	0.0315 (9)	
C2	0.56213 (18)	−0.3272 (5)	0.9617 (2)	0.0308 (9)	
C20	1.09503 (18)	0.1988 (5)	0.8674 (2)	0.0309 (9)	
C11	0.68606 (18)	0.2301 (5)	0.6354 (2)	0.0311 (9)	
C9	0.6682 (2)	0.5311 (5)	0.6731 (2)	0.0345 (10)	
H9A	0.7051	0.5634	0.6524	0.041*	
H9B	0.6796	0.5166	0.7195	0.041*	
C27	0.82314 (18)	0.2433 (5)	0.8709 (2)	0.0315 (9)	
C3	0.60166 (18)	−0.2074 (5)	0.93082 (19)	0.0301 (9)	
C25	0.82936 (19)	−0.0520 (5)	0.8253 (2)	0.0341 (10)	
H25A	0.8201	−0.0296	0.7789	0.041*	
H25B	0.7907	−0.0736	0.8443	0.041*	
C19	1.08842 (19)	0.1695 (5)	0.93181 (19)	0.0316 (9)	
C23	0.96153 (19)	−0.0141 (6)	0.8346 (2)	0.0357 (10)	
H23A	0.9982	0.0104	0.8123	0.043*	
H23B	0.9742	−0.0317	0.8806	0.043*	
C21	1.1136 (2)	0.0116 (6)	0.9694 (2)	0.0377 (10)	
H21A	1.1044	−0.0924	0.9433	0.045*	
H21B	1.0928	0.0007	1.0091	0.045*	
C28	0.7616 (2)	0.2637 (6)	0.8454 (2)	0.0397 (11)	
H28	0.7448	0.1857	0.8139	0.048*	
C10	0.62039 (19)	0.6734 (5)	0.6625 (2)	0.0336 (10)	
H10A	0.6365	0.7809	0.6825	0.040*	
H10B	0.6116	0.6941	0.6161	0.040*	
C5	0.6318 (2)	−0.0569 (5)	0.9687 (2)	0.0359 (10)	
H5A	0.6721	−0.0353	0.9533	0.043*	
H5B	0.6381	−0.0893	1.0145	0.043*	
C7	0.53832 (19)	0.4524 (6)	0.6672 (2)	0.0378 (10)	
H7A	0.5248	0.4610	0.6211	0.045*	
H7B	0.5029	0.4196	0.6903	0.045*	
C16	0.74788 (19)	0.2396 (6)	0.6587 (2)	0.0357 (10)	
H16	0.7618	0.3334	0.6849	0.043*	
C15	0.7893 (2)	0.1105 (6)	0.6436 (2)	0.0412 (11)	
H15	0.8308	0.1192	0.6592	0.049*	
C26	0.87103 (19)	−0.2105 (6)	0.8348 (2)	0.0371 (10)	
H26A	0.8784	−0.2368	0.8811	0.044*	
H26B	0.8511	−0.3110	0.8132	0.044*	
C12	0.6660 (2)	0.0859 (6)	0.5972 (2)	0.0369 (10)	
H12	0.6246	0.0765	0.5812	0.044*	
C8	0.58805 (18)	0.3138 (5)	0.6776 (2)	0.0344 (10)	
H8A	0.5991	0.2979	0.7240	0.041*	
H8B	0.5724	0.2033	0.6597	0.041*	
C32	0.8473 (2)	0.3663 (5)	0.9167 (2)	0.0374 (10)	
H32	0.8884	0.3573	0.9337	0.045*	
C24	0.91766 (19)	0.1391 (6)	0.8266 (2)	0.0362 (10)	
H24A	0.9370	0.2417	0.8473	0.043*	
H24B	0.9083	0.1645	0.7805	0.043*	
C29	0.7254 (2)	0.3984 (7)	0.8662 (2)	0.0480 (12)	
H29	0.6843	0.4089	0.8493	0.058*	
C31	0.8113 (2)	0.4999 (6)	0.9369 (2)	0.0442 (12)	
H31	0.8282	0.5796	0.9677	0.053*	
C13	0.7077 (2)	−0.0424 (6)	0.5832 (2)	0.0423 (11)	
H13	0.6938	−0.1384	0.5582	0.051*	
C22	1.1830 (2)	0.0208 (6)	0.9874 (2)	0.0433 (11)	
H22A	1.1963	−0.0826	1.0111	0.065*	
H22B	1.2040	0.0288	0.9483	0.065*	
H22C	1.1924	0.1219	1.0141	0.065*	
C14	0.7692 (2)	−0.0311 (6)	0.6054 (2)	0.0450 (12)	
H14	0.7970	−0.1173	0.5948	0.054*	
C30	0.7503 (2)	0.5173 (6)	0.9120 (2)	0.0487 (13)	
H30	0.7261	0.6082	0.9259	0.058*	
C6	0.5943 (2)	0.1094 (6)	0.9627 (2)	0.0529 (13)	
H6C	0.6158	0.2002	0.9878	0.079*	
H6D	0.5887	0.1440	0.9177	0.079*	
H6E	0.5546	0.0899	0.9788	0.079*	

### (I) 4-Phenylpiperazin-1-ium 6-chloro-5-ethyl-2,4-dioxopyrimidin-1-ide Atomic displacement parameters (Å^2^)

**Table:**

	*U*^11^	*U*^22^	*U*^33^	*U*^12^	*U*^13^	*U*^23^
Cl2	0.0470 (6)	0.0405 (6)	0.0359 (6)	0.0097 (5)	0.0099 (5)	−0.0029 (5)
Cl1	0.0503 (7)	0.0474 (7)	0.0347 (6)	−0.0174 (5)	0.0101 (5)	0.0010 (5)
O1	0.0488 (18)	0.0360 (17)	0.0301 (16)	−0.0110 (14)	0.0063 (14)	−0.0051 (13)
O2	0.0396 (17)	0.0410 (17)	0.0226 (15)	−0.0075 (13)	0.0030 (13)	0.0008 (12)
O3	0.0446 (18)	0.0400 (18)	0.0299 (16)	0.0085 (14)	0.0087 (14)	0.0022 (13)
N1	0.0337 (18)	0.0299 (18)	0.0231 (17)	−0.0075 (15)	0.0036 (14)	0.0018 (14)
O4	0.0462 (19)	0.0444 (19)	0.0349 (17)	0.0130 (15)	0.0087 (14)	0.0079 (14)
N7	0.0323 (18)	0.033 (2)	0.0266 (18)	0.0058 (15)	0.0057 (15)	−0.0034 (15)
N2	0.0301 (18)	0.036 (2)	0.0244 (17)	−0.0008 (15)	0.0033 (14)	0.0004 (15)
N8	0.0331 (19)	0.036 (2)	0.0305 (19)	0.0011 (16)	0.0053 (15)	−0.0007 (16)
N3	0.0349 (19)	0.0336 (19)	0.0280 (18)	0.0064 (15)	0.0052 (15)	−0.0001 (15)
N4	0.0304 (19)	0.033 (2)	0.0355 (19)	−0.0054 (15)	0.0046 (15)	−0.0023 (16)
N5	0.0297 (18)	0.0307 (19)	0.038 (2)	0.0003 (15)	0.0089 (16)	−0.0023 (16)
N6	0.038 (2)	0.035 (2)	0.0287 (19)	0.0004 (16)	0.0042 (16)	−0.0002 (15)
C1	0.032 (2)	0.033 (2)	0.028 (2)	−0.0028 (18)	0.0043 (18)	0.0009 (18)
C18	0.035 (2)	0.032 (2)	0.032 (2)	−0.0007 (18)	0.0079 (19)	0.0012 (18)
C17	0.032 (2)	0.035 (2)	0.028 (2)	0.0040 (18)	0.0048 (18)	0.0007 (18)
C4	0.027 (2)	0.035 (2)	0.033 (2)	−0.0055 (17)	0.0018 (18)	0.0043 (18)
C2	0.029 (2)	0.031 (2)	0.032 (2)	0.0002 (17)	0.0011 (18)	0.0027 (18)
C20	0.028 (2)	0.033 (2)	0.032 (2)	0.0001 (18)	0.0054 (18)	−0.0055 (18)
C11	0.029 (2)	0.033 (2)	0.032 (2)	0.0018 (18)	0.0075 (18)	0.0018 (18)
C9	0.039 (2)	0.029 (2)	0.036 (2)	−0.0052 (19)	0.009 (2)	−0.0006 (19)
C27	0.031 (2)	0.033 (2)	0.031 (2)	−0.0006 (18)	0.0070 (18)	0.0058 (18)
C3	0.031 (2)	0.033 (2)	0.026 (2)	−0.0006 (17)	−0.0008 (17)	0.0012 (17)
C25	0.032 (2)	0.038 (2)	0.032 (2)	−0.0057 (19)	0.0044 (18)	0.0012 (19)
C19	0.034 (2)	0.032 (2)	0.029 (2)	0.0009 (18)	0.0051 (18)	0.0000 (18)
C23	0.031 (2)	0.039 (3)	0.038 (2)	−0.0023 (19)	0.0063 (19)	−0.006 (2)
C21	0.042 (3)	0.038 (2)	0.035 (2)	0.006 (2)	0.010 (2)	0.004 (2)
C28	0.039 (2)	0.044 (3)	0.037 (3)	0.004 (2)	0.006 (2)	−0.001 (2)
C10	0.041 (2)	0.033 (2)	0.027 (2)	0.0013 (19)	0.0050 (19)	0.0011 (18)
C5	0.041 (2)	0.039 (3)	0.028 (2)	−0.009 (2)	0.0027 (19)	0.0014 (19)
C7	0.031 (2)	0.044 (3)	0.038 (2)	0.004 (2)	0.0009 (19)	−0.003 (2)
C16	0.034 (2)	0.038 (2)	0.036 (2)	0.0002 (19)	0.0054 (19)	−0.0014 (19)
C15	0.036 (2)	0.045 (3)	0.043 (3)	0.006 (2)	0.008 (2)	0.004 (2)
C26	0.038 (2)	0.039 (3)	0.034 (2)	−0.006 (2)	0.007 (2)	−0.0022 (19)
C12	0.040 (2)	0.037 (2)	0.034 (2)	0.000 (2)	0.0024 (19)	0.0026 (19)
C8	0.035 (2)	0.031 (2)	0.038 (2)	−0.0043 (19)	0.0045 (19)	−0.0033 (19)
C32	0.039 (2)	0.033 (2)	0.041 (3)	−0.0047 (19)	0.011 (2)	−0.002 (2)
C24	0.032 (2)	0.041 (3)	0.037 (2)	−0.0063 (19)	0.0111 (19)	−0.002 (2)
C29	0.044 (3)	0.055 (3)	0.045 (3)	0.015 (2)	0.006 (2)	0.005 (2)
C31	0.055 (3)	0.037 (3)	0.043 (3)	−0.001 (2)	0.017 (2)	−0.003 (2)
C13	0.058 (3)	0.034 (2)	0.037 (3)	−0.004 (2)	0.014 (2)	−0.006 (2)
C22	0.042 (3)	0.046 (3)	0.043 (3)	0.005 (2)	0.007 (2)	0.009 (2)
C14	0.053 (3)	0.037 (3)	0.047 (3)	0.010 (2)	0.017 (2)	0.002 (2)
C30	0.056 (3)	0.045 (3)	0.047 (3)	0.017 (2)	0.019 (3)	0.005 (2)
C6	0.065 (3)	0.040 (3)	0.051 (3)	0.002 (2)	−0.008 (3)	−0.010 (2)

### (I) 4-Phenylpiperazin-1-ium 6-chloro-5-ethyl-2,4-dioxopyrimidin-1-ide Geometric parameters (Å, º)

**Table:**

Cl2—C20	1.758 (4)	C19—C21	1.509 (6)
Cl1—C4	1.747 (4)	C23—C24	1.508 (6)
O1—C1	1.249 (5)	C23—H23A	0.9700
O2—C2	1.248 (5)	C23—H23B	0.9700
O3—C18	1.249 (5)	C21—C22	1.517 (6)
N1—C1	1.382 (5)	C21—H21A	0.9700
N1—C2	1.384 (5)	C21—H21B	0.9700
N1—H1	0.8600	C28—C29	1.386 (6)
O4—C17	1.243 (5)	C28—H28	0.9300
N7—C17	1.380 (5)	C10—H10A	0.9700
N7—C18	1.388 (5)	C10—H10B	0.9700
N7—H7	0.8600	C5—C6	1.508 (6)
N2—C1	1.347 (5)	C5—H5A	0.9700
N2—C4	1.351 (5)	C5—H5B	0.9700
N8—C20	1.342 (5)	C7—C8	1.513 (6)
N8—C17	1.354 (5)	C7—H7A	0.9700
N3—C7	1.478 (5)	C7—H7B	0.9700
N3—C10	1.487 (5)	C16—C15	1.388 (6)
N3—H3A	0.8900	C16—H16	0.9300
N3—H3B	0.8900	C15—C14	1.385 (7)
N4—C11	1.419 (5)	C15—H15	0.9300
N4—C9	1.467 (5)	C26—H26A	0.9700
N4—C8	1.475 (5)	C26—H26B	0.9700
N5—C27	1.410 (5)	C12—C13	1.380 (6)
N5—C25	1.454 (5)	C12—H12	0.9300
N5—C24	1.468 (5)	C8—H8A	0.9700
N6—C23	1.482 (5)	C8—H8B	0.9700
N6—C26	1.495 (5)	C32—C31	1.371 (6)
N6—H6A	0.8900	C32—H32	0.9300
N6—H6B	0.8900	C24—H24A	0.9700
C18—C19	1.436 (6)	C24—H24B	0.9700
C4—C3	1.359 (6)	C29—C30	1.383 (7)
C2—C3	1.440 (6)	C29—H29	0.9300
C20—C19	1.362 (6)	C31—C30	1.383 (7)
C11—C16	1.385 (6)	C31—H31	0.9300
C11—C12	1.400 (6)	C13—C14	1.374 (7)
C9—C10	1.505 (6)	C13—H13	0.9300
C9—H9A	0.9700	C22—H22A	0.9600
C9—H9B	0.9700	C22—H22B	0.9600
C27—C28	1.398 (6)	C22—H22C	0.9600
C27—C32	1.399 (6)	C14—H14	0.9300
C3—C5	1.505 (6)	C30—H30	0.9300
C25—C26	1.514 (6)	C6—H6C	0.9600
C25—H25A	0.9700	C6—H6D	0.9600
C25—H25B	0.9700	C6—H6E	0.9600
			
C1—N1—C2	125.1 (3)	H21A—C21—H21B	107.8
C1—N1—H1	117.5	C29—C28—C27	121.0 (5)
C2—N1—H1	117.5	C29—C28—H28	119.5
C17—N7—C18	125.6 (3)	C27—C28—H28	119.5
C17—N7—H7	117.2	N3—C10—C9	110.7 (3)
C18—N7—H7	117.2	N3—C10—H10A	109.5
C1—N2—C4	117.3 (3)	C9—C10—H10A	109.5
C20—N8—C17	117.0 (3)	N3—C10—H10B	109.5
C7—N3—C10	112.2 (3)	C9—C10—H10B	109.5
C7—N3—H3A	109.2	H10A—C10—H10B	108.1
C10—N3—H3A	109.2	C3—C5—C6	113.3 (4)
C7—N3—H3B	109.2	C3—C5—H5A	108.9
C10—N3—H3B	109.2	C6—C5—H5A	108.9
H3A—N3—H3B	107.9	C3—C5—H5B	108.9
C11—N4—C9	117.6 (3)	C6—C5—H5B	108.9
C11—N4—C8	115.6 (3)	H5A—C5—H5B	107.7
C9—N4—C8	110.1 (3)	N3—C7—C8	110.3 (3)
C27—N5—C25	117.8 (3)	N3—C7—H7A	109.6
C27—N5—C24	116.5 (3)	C8—C7—H7A	109.6
C25—N5—C24	110.9 (3)	N3—C7—H7B	109.6
C23—N6—C26	112.2 (3)	C8—C7—H7B	109.6
C23—N6—H6A	109.2	H7A—C7—H7B	108.1
C26—N6—H6A	109.2	C11—C16—C15	120.8 (4)
C23—N6—H6B	109.2	C11—C16—H16	119.6
C26—N6—H6B	109.2	C15—C16—H16	119.6
H6A—N6—H6B	107.9	C14—C15—C16	120.3 (4)
O1—C1—N2	121.5 (4)	C14—C15—H15	119.8
O1—C1—N1	120.3 (4)	C16—C15—H15	119.8
N2—C1—N1	118.2 (4)	N6—C26—C25	109.6 (3)
O3—C18—N7	119.1 (4)	N6—C26—H26A	109.8
O3—C18—C19	125.1 (4)	C25—C26—H26A	109.8
N7—C18—C19	115.8 (3)	N6—C26—H26B	109.8
O4—C17—N8	121.8 (4)	C25—C26—H26B	109.8
O4—C17—N7	120.4 (4)	H26A—C26—H26B	108.2
N8—C17—N7	117.8 (4)	C13—C12—C11	120.0 (4)
N2—C4—C3	128.3 (4)	C13—C12—H12	120.0
N2—C4—Cl1	112.1 (3)	C11—C12—H12	120.0
C3—C4—Cl1	119.5 (3)	N4—C8—C7	110.1 (3)
O2—C2—N1	119.5 (4)	N4—C8—H8A	109.6
O2—C2—C3	124.5 (4)	C7—C8—H8A	109.6
N1—C2—C3	116.1 (3)	N4—C8—H8B	109.6
N8—C20—C19	129.3 (4)	C7—C8—H8B	109.6
N8—C20—Cl2	111.7 (3)	H8A—C8—H8B	108.2
C19—C20—Cl2	119.0 (3)	C31—C32—C27	121.1 (4)
C16—C11—C12	118.5 (4)	C31—C32—H32	119.5
C16—C11—N4	122.4 (4)	C27—C32—H32	119.5
C12—C11—N4	118.9 (4)	N5—C24—C23	110.1 (3)
N4—C9—C10	109.9 (4)	N5—C24—H24A	109.6
N4—C9—H9A	109.7	C23—C24—H24A	109.6
C10—C9—H9A	109.7	N5—C24—H24B	109.6
N4—C9—H9B	109.7	C23—C24—H24B	109.6
C10—C9—H9B	109.7	H24A—C24—H24B	108.1
H9A—C9—H9B	108.2	C30—C29—C28	120.1 (5)
C28—C27—C32	117.7 (4)	C30—C29—H29	120.0
C28—C27—N5	123.4 (4)	C28—C29—H29	120.0
C32—C27—N5	118.8 (4)	C32—C31—C30	120.7 (5)
C4—C3—C2	115.0 (4)	C32—C31—H31	119.6
C4—C3—C5	124.6 (4)	C30—C31—H31	119.6
C2—C3—C5	120.4 (3)	C14—C13—C12	121.3 (4)
N5—C25—C26	109.5 (4)	C14—C13—H13	119.3
N5—C25—H25A	109.8	C12—C13—H13	119.3
C26—C25—H25A	109.8	C21—C22—H22A	109.5
N5—C25—H25B	109.8	C21—C22—H22B	109.5
C26—C25—H25B	109.8	H22A—C22—H22B	109.5
H25A—C25—H25B	108.2	C21—C22—H22C	109.5
C20—C19—C18	114.5 (4)	H22A—C22—H22C	109.5
C20—C19—C21	124.4 (4)	H22B—C22—H22C	109.5
C18—C19—C21	121.1 (4)	C13—C14—C15	119.0 (4)
N6—C23—C24	110.4 (4)	C13—C14—H14	120.5
N6—C23—H23A	109.6	C15—C14—H14	120.5
C24—C23—H23A	109.6	C29—C30—C31	119.4 (4)
N6—C23—H23B	109.6	C29—C30—H30	120.3
C24—C23—H23B	109.6	C31—C30—H30	120.3
H23A—C23—H23B	108.1	C5—C6—H6C	109.5
C19—C21—C22	113.2 (4)	C5—C6—H6D	109.5
C19—C21—H21A	108.9	H6C—C6—H6D	109.5
C22—C21—H21A	108.9	C5—C6—H6E	109.5
C19—C21—H21B	108.9	H6C—C6—H6E	109.5
C22—C21—H21B	108.9	H6D—C6—H6E	109.5
			
C4—N2—C1—O1	179.0 (4)	N8—C20—C19—C21	−179.6 (4)
C4—N2—C1—N1	−0.6 (6)	Cl2—C20—C19—C21	2.8 (6)
C2—N1—C1—O1	−178.9 (4)	O3—C18—C19—C20	178.5 (4)
C2—N1—C1—N2	0.7 (6)	N7—C18—C19—C20	−1.4 (6)
C17—N7—C18—O3	−179.1 (4)	O3—C18—C19—C21	−0.9 (7)
C17—N7—C18—C19	0.9 (6)	N7—C18—C19—C21	179.2 (4)
C20—N8—C17—O4	179.6 (4)	C26—N6—C23—C24	−53.9 (5)
C20—N8—C17—N7	−0.8 (6)	C20—C19—C21—C22	76.2 (6)
C18—N7—C17—O4	179.9 (4)	C18—C19—C21—C22	−104.4 (5)
C18—N7—C17—N8	0.3 (6)	C32—C27—C28—C29	−1.8 (6)
C1—N2—C4—C3	1.1 (7)	N5—C27—C28—C29	173.5 (4)
C1—N2—C4—Cl1	−177.7 (3)	C7—N3—C10—C9	−54.1 (4)
C1—N1—C2—O2	179.0 (4)	N4—C9—C10—N3	56.9 (4)
C1—N1—C2—C3	−1.0 (6)	C4—C3—C5—C6	84.3 (5)
C17—N8—C20—C19	0.1 (7)	C2—C3—C5—C6	−92.5 (5)
C17—N8—C20—Cl2	177.9 (3)	C10—N3—C7—C8	53.7 (4)
C9—N4—C11—C16	8.8 (6)	C12—C11—C16—C15	1.2 (6)
C8—N4—C11—C16	−124.1 (4)	N4—C11—C16—C15	−174.7 (4)
C9—N4—C11—C12	−167.1 (4)	C11—C16—C15—C14	−0.7 (7)
C8—N4—C11—C12	60.0 (5)	C23—N6—C26—C25	54.9 (5)
C11—N4—C9—C10	164.1 (3)	N5—C25—C26—N6	−58.0 (4)
C8—N4—C9—C10	−60.6 (4)	C16—C11—C12—C13	−0.4 (6)
C25—N5—C27—C28	−13.7 (6)	N4—C11—C12—C13	175.6 (4)
C24—N5—C27—C28	121.7 (4)	C11—N4—C8—C7	−163.1 (4)
C25—N5—C27—C32	161.5 (4)	C9—N4—C8—C7	60.6 (4)
C24—N5—C27—C32	−63.1 (5)	N3—C7—C8—N4	−56.5 (4)
N2—C4—C3—C2	−1.4 (7)	C28—C27—C32—C31	1.5 (6)
Cl1—C4—C3—C2	177.3 (3)	N5—C27—C32—C31	−174.0 (4)
N2—C4—C3—C5	−178.3 (4)	C27—N5—C24—C23	161.1 (4)
Cl1—C4—C3—C5	0.4 (6)	C25—N5—C24—C23	−60.5 (5)
O2—C2—C3—C4	−178.8 (4)	N6—C23—C24—N5	55.5 (4)
N1—C2—C3—C4	1.2 (6)	C27—C28—C29—C30	1.2 (7)
O2—C2—C3—C5	−1.7 (6)	C27—C32—C31—C30	−0.6 (7)
N1—C2—C3—C5	178.3 (4)	C11—C12—C13—C14	−0.8 (7)
C27—N5—C25—C26	−160.5 (3)	C12—C13—C14—C15	1.3 (7)
C24—N5—C25—C26	61.7 (4)	C16—C15—C14—C13	−0.5 (7)
N8—C20—C19—C18	1.1 (7)	C28—C29—C30—C31	−0.3 (7)
Cl2—C20—C19—C18	−176.5 (3)	C32—C31—C30—C29	0.0 (7)

### (I) 4-Phenylpiperazin-1-ium 6-chloro-5-ethyl-2,4-dioxopyrimidin-1-ide Hydrogen-bond geometry (Å, º)

**Table:**

*D*—H···*A*	*D*—H	H···*A*	*D*···*A*	*D*—H···*A*
N1—H1···O2^i^	0.86	2.00	2.859 (4)	173
N3—H3*A*···O1^ii^	0.89	2.83	3.465 (4)	129
N6—H6*A*···O4^iii^	0.89	1.81	2.681 (5)	165
N7—H7···O3^iv^	0.86	2.02	2.873 (4)	174
N3—H3*A*···N2^ii^	0.89	1.92	2.808 (4)	174
N6—H6*B*···N8^v^	0.89	1.92	2.798 (5)	169
C10—H10*B*···O2^vi^	0.97	2.46	3.355 (5)	154
C26—H26*A*···O3^vii^	0.97	2.58	3.444 (5)	147
C16—H16···Cl2^viii^	0.93	2.80	3.462 (4)	129

Symmetry codes: (i) −*x*+1, −*y*−1, −*z*+2; (ii) *x*, *y*+1, *z*; (iii) *x*, *y*−1, *z*; (iv) −*x*+2, −*y*+1, −*z*+2; (v) −*x*+2, *y*−1/2, −*z*+3/2; (vi) *x*, −*y*+1/2, *z*−1/2; (vii) −*x*+2, −*y*, −*z*+2; (viii) −*x*+2, *y*+1/2, −*z*+3/2.

### (II) 4-Phenylpiperazin-1-ium 6-chloro-5-isopropyl-2,4-dioxopyrimidin-1-ide Crystal data

**Table:**

C_10_H_15_N_2_^+^·C_7_H_8_ClN_2_O_2_^−^	*F*(000) = 1488
*M**_r_* = 350.84	*D*_x_ = 1.318 Mg m^−^^3^
Monoclinic, *I*2/*a*	Cu *K*α radiation, λ = 1.5418 Å
*a* = 20.5012 (3) Å	Cell parameters from 6927 reflections
*b* = 7.4565 (1) Å	θ = 3.8–70.0°
*c* = 23.1414 (3) Å	µ = 2.05 mm^−^^1^
β = 90.639 (1)°	*T* = 101 K
*V* = 3537.34 (8) Å^3^	Prism, colourless
*Z* = 8	0.34 × 0.13 × 0.09 mm

### (II) 4-Phenylpiperazin-1-ium 6-chloro-5-isopropyl-2,4-dioxopyrimidin-1-ide Data collection

**Table:**

Agilent Xcalibur Ruby Gemini diffractometer	2926 reflections with *I* > 2σ(*I*)
Detector resolution: 10.2673 pixels mm^-1^	*R*_int_ = 0.069
ω scans	θ_max_ = 70.6°, θ_min_ = 3.8°
Absorption correction: multi-scan (CrysAlis PRO; Agilent, 2014)	*h* = −24→25
*T*_min_ = 0.760, *T*_max_ = 0.828	*k* = −9→7
13174 measured reflections	*l* = −27→28
3396 independent reflections	

### (II) 4-Phenylpiperazin-1-ium 6-chloro-5-isopropyl-2,4-dioxopyrimidin-1-ide Refinement

**Table:**

Refinement on *F*^2^	0 restraints
Least-squares matrix: full	Hydrogen site location: inferred from neighbouring sites
*R*[*F*^2^ > 2σ(*F*^2^)] = 0.044	H-atom parameters constrained
*wR*(*F*^2^) = 0.122	*w* = 1/[σ^2^(*F*_o_^2^) + (0.0585*P*)^2^ + 3.6877*P*] where *P* = (*F*_o_^2^ + 2*F*_c_^2^)/3
*S* = 1.03	(Δ/σ)_max_ < 0.001
3346 reflections	Δρ_max_ = 0.55 e Å^−^^3^
217 parameters	Δρ_min_ = −0.56 e Å^−^^3^

### (II) 4-Phenylpiperazin-1-ium 6-chloro-5-isopropyl-2,4-dioxopyrimidin-1-ide Special details

**Table:**

Geometry. All e.s.d.'s (except the e.s.d. in the dihedral angle between two l.s. planes) are estimated using the full covariance matrix. The cell e.s.d.'s are taken into account individually in the estimation of e.s.d.'s in distances, angles and torsion angles; correlations between e.s.d.'s in cell parameters are only used when they are defined by crystal symmetry. An approximate (isotropic) treatment of cell e.s.d.'s is used for estimating e.s.d.'s involving l.s. planes.

### (II) 4-Phenylpiperazin-1-ium 6-chloro-5-isopropyl-2,4-dioxopyrimidin-1-ide Fractional atomic coordinates and isotropic or equivalent isotropic displacement parameters (Å^2^)

**Table:**

	*x*	*y*	*z*	*U*_iso_*/*U*_eq_	
Cl1	0.43825 (2)	0.80800 (7)	0.38729 (2)	0.03420 (17)	
O1	0.42855 (6)	1.32956 (18)	0.26398 (6)	0.0286 (3)	
O2	0.23184 (6)	1.07264 (19)	0.29852 (6)	0.0288 (3)	
N3	0.33106 (7)	1.1946 (2)	0.28125 (6)	0.0227 (3)	
H3	0.3124	1.2711	0.2587	0.027*	
N4	0.42771 (7)	1.0889 (2)	0.32365 (6)	0.0228 (3)	
N2	0.56397 (7)	1.1049 (2)	0.31180 (6)	0.0246 (3)	
H3N	0.5734	1.0238	0.2849	0.029*	
H2N	0.5208	1.1087	0.3152	0.029*	
C11	0.39765 (9)	1.2092 (2)	0.28906 (7)	0.0228 (4)	
N1	0.60877 (7)	1.3622 (2)	0.39443 (6)	0.0230 (3)	
C2	0.58171 (9)	1.1911 (2)	0.41332 (8)	0.0237 (4)	
H2A	0.5352	1.2036	0.4195	0.028*	
H2B	0.6020	1.1556	0.4496	0.028*	
C4	0.58796 (10)	1.2834 (3)	0.29310 (8)	0.0271 (4)	
H4A	0.5649	1.3201	0.2582	0.032*	
H4B	0.6341	1.2760	0.2845	0.032*	
C14	0.39071 (9)	0.9583 (2)	0.34646 (7)	0.0236 (4)	
C12	0.29194 (9)	1.0666 (3)	0.30679 (7)	0.0232 (4)	
C6	0.58544 (9)	1.4868 (3)	0.49109 (8)	0.0273 (4)	
H6	0.5581	1.3906	0.4992	0.033*	
C13	0.32496 (9)	0.9324 (2)	0.34113 (7)	0.0231 (4)	
C3	0.59378 (9)	1.0490 (3)	0.36799 (8)	0.0245 (4)	
H3A	0.6403	1.0318	0.3634	0.029*	
H3B	0.5750	0.9361	0.3802	0.029*	
C10	0.65511 (9)	1.6458 (3)	0.42609 (8)	0.0272 (4)	
H10	0.6749	1.6571	0.3903	0.033*	
C15	0.28407 (10)	0.7802 (3)	0.36508 (8)	0.0286 (4)	
H15	0.2393	0.8063	0.3523	0.034*	
C5	0.61534 (9)	1.4980 (2)	0.43717 (8)	0.0224 (4)	
C8	0.63624 (10)	1.7627 (3)	0.52142 (9)	0.0341 (5)	
H8	0.6436	1.8500	0.5494	0.041*	
C1	0.57729 (9)	1.4207 (3)	0.34016 (7)	0.0249 (4)	
H1A	0.5953	1.5352	0.3284	0.030*	
H1B	0.5309	1.4365	0.3462	0.030*	
C7	0.59623 (10)	1.6182 (3)	0.53262 (8)	0.0332 (5)	
H7	0.5763	1.6088	0.5684	0.040*	
C9	0.66531 (10)	1.7757 (3)	0.46790 (9)	0.0320 (5)	
H9	0.6921	1.8731	0.4599	0.038*	
C16	0.28114 (10)	0.7739 (3)	0.43025 (8)	0.0310 (4)	
H16A	0.2708	0.8909	0.4448	0.047*	
H16B	0.2481	0.6902	0.4418	0.047*	
H16C	0.3227	0.7365	0.4456	0.047*	
C17	0.30027 (12)	0.6004 (3)	0.33815 (9)	0.0394 (5)	
H17A	0.3015	0.6125	0.2969	0.059*	
H17B	0.3421	0.5605	0.3522	0.059*	
H17C	0.2675	0.5143	0.3483	0.059*	

### (II) 4-Phenylpiperazin-1-ium 6-chloro-5-isopropyl-2,4-dioxopyrimidin-1-ide Atomic displacement parameters (Å^2^)

**Table:**

	*U*^11^	*U*^22^	*U*^33^	*U*^12^	*U*^13^	*U*^23^
Cl1	0.0307 (3)	0.0306 (3)	0.0413 (3)	0.00185 (19)	0.0001 (2)	0.0141 (2)
O1	0.0225 (7)	0.0278 (7)	0.0355 (7)	−0.0043 (6)	0.0020 (5)	0.0107 (6)
O2	0.0210 (6)	0.0341 (8)	0.0313 (7)	−0.0042 (6)	0.0017 (5)	0.0053 (6)
N3	0.0210 (7)	0.0233 (8)	0.0238 (7)	−0.0013 (6)	0.0012 (6)	0.0032 (6)
N4	0.0233 (7)	0.0211 (8)	0.0239 (7)	0.0003 (6)	0.0022 (6)	0.0025 (6)
N2	0.0227 (7)	0.0256 (8)	0.0255 (7)	0.0009 (7)	0.0023 (6)	−0.0055 (6)
C11	0.0238 (9)	0.0216 (9)	0.0231 (8)	−0.0010 (7)	0.0033 (7)	−0.0021 (7)
N1	0.0261 (8)	0.0203 (8)	0.0225 (7)	0.0005 (6)	0.0007 (6)	0.0002 (6)
C2	0.0254 (9)	0.0221 (9)	0.0235 (8)	0.0009 (7)	0.0020 (7)	0.0010 (7)
C4	0.0302 (10)	0.0284 (10)	0.0226 (9)	−0.0025 (8)	0.0023 (7)	−0.0003 (8)
C14	0.0295 (9)	0.0207 (9)	0.0206 (8)	0.0023 (8)	0.0024 (7)	−0.0001 (7)
C12	0.0255 (9)	0.0244 (9)	0.0198 (8)	−0.0036 (8)	0.0043 (7)	−0.0030 (7)
C6	0.0300 (10)	0.0248 (10)	0.0273 (9)	−0.0002 (8)	0.0003 (8)	−0.0004 (8)
C13	0.0287 (9)	0.0209 (9)	0.0198 (8)	−0.0031 (8)	0.0042 (7)	−0.0022 (7)
C3	0.0231 (9)	0.0221 (9)	0.0284 (9)	0.0017 (7)	0.0017 (7)	−0.0005 (7)
C10	0.0241 (9)	0.0254 (10)	0.0322 (10)	0.0019 (8)	0.0017 (7)	−0.0005 (8)
C15	0.0317 (10)	0.0262 (10)	0.0279 (9)	−0.0059 (8)	0.0039 (8)	0.0007 (8)
C5	0.0201 (8)	0.0211 (9)	0.0260 (9)	0.0053 (7)	−0.0028 (7)	−0.0015 (7)
C8	0.0376 (11)	0.0290 (11)	0.0354 (10)	0.0050 (9)	−0.0084 (9)	−0.0106 (9)
C1	0.0284 (9)	0.0236 (9)	0.0227 (8)	0.0002 (8)	0.0006 (7)	0.0016 (7)
C7	0.0385 (11)	0.0350 (11)	0.0260 (9)	0.0066 (9)	0.0012 (8)	−0.0053 (8)
C9	0.0273 (10)	0.0241 (10)	0.0445 (11)	−0.0005 (8)	−0.0056 (8)	−0.0034 (9)
C16	0.0318 (10)	0.0306 (11)	0.0309 (10)	−0.0049 (9)	0.0092 (8)	0.0003 (8)
C17	0.0492 (13)	0.0299 (11)	0.0396 (11)	−0.0130 (10)	0.0139 (10)	−0.0048 (9)

### (II) 4-Phenylpiperazin-1-ium 6-chloro-5-isopropyl-2,4-dioxopyrimidin-1-ide Geometric parameters (Å, º)

**Table:**

Cl1—C14	1.7544 (18)	C6—C7	1.389 (3)
O1—C11	1.246 (2)	C6—C5	1.399 (3)
O2—C12	1.246 (2)	C6—H6	0.9300
N3—C11	1.379 (2)	C13—C15	1.519 (3)
N3—C12	1.383 (2)	C3—H3A	0.9700
N3—H3	0.8600	C3—H3B	0.9700
N4—N4	0.000 (5)	C10—C9	1.383 (3)
N4—C14	1.346 (2)	C10—C5	1.397 (3)
N4—C11	1.347 (2)	C10—H10	0.9300
N2—C4	1.485 (2)	C15—C16	1.511 (3)
N2—C3	1.490 (2)	C15—C17	1.517 (3)
N2—H3N	0.8900	C15—H15	0.9800
N2—H2N	0.8900	C8—C7	1.380 (3)
C11—N4	1.347 (2)	C8—C9	1.384 (3)
N1—C5	1.421 (2)	C8—H8	0.9300
N1—C2	1.460 (2)	C1—H1A	0.9700
N1—C1	1.472 (2)	C1—H1B	0.9700
C2—C3	1.513 (2)	C7—H7	0.9300
C2—H2A	0.9700	C9—H9	0.9300
C2—H2B	0.9700	C16—H16A	0.9600
C4—C1	1.512 (3)	C16—H16B	0.9600
C4—H4A	0.9700	C16—H16C	0.9600
C4—H4B	0.9700	C17—H17A	0.9600
C14—N4	1.346 (2)	C17—H17B	0.9600
C14—C13	1.366 (3)	C17—H17C	0.9600
C12—C13	1.442 (3)		
			
C11—N3—C12	125.17 (16)	C12—C13—C15	117.51 (16)
C11—N3—H3	117.4	N2—C3—C2	109.92 (15)
C12—N3—H3	117.4	N2—C3—H3A	109.7
N4—N4—C14	0 (10)	C2—C3—H3A	109.7
N4—N4—C11	0 (10)	N2—C3—H3B	109.7
C14—N4—C11	117.31 (15)	C2—C3—H3B	109.7
C4—N2—C3	111.77 (14)	H3A—C3—H3B	108.2
C4—N2—H3N	109.3	C9—C10—C5	120.54 (18)
C3—N2—H3N	109.3	C9—C10—H10	119.7
C4—N2—H2N	109.3	C5—C10—H10	119.7
C3—N2—H2N	109.3	C16—C15—C17	113.20 (18)
H3N—N2—H2N	107.9	C16—C15—C13	114.57 (16)
O1—C11—N4	121.67 (16)	C17—C15—C13	112.72 (16)
O1—C11—N4	121.67 (16)	C16—C15—H15	105.1
N4—C11—N4	0.00 (14)	C17—C15—H15	105.1
O1—C11—N3	120.23 (17)	C13—C15—H15	105.1
N4—C11—N3	118.10 (16)	C10—C5—C6	118.26 (17)
N4—C11—N3	118.10 (16)	C10—C5—N1	119.06 (16)
C5—N1—C2	116.59 (14)	C6—C5—N1	122.64 (17)
C5—N1—C1	114.83 (15)	C7—C8—C9	119.04 (19)
C2—N1—C1	110.50 (14)	C7—C8—H8	120.5
N1—C2—C3	109.78 (14)	C9—C8—H8	120.5
N1—C2—H2A	109.7	N1—C1—C4	110.37 (15)
C3—C2—H2A	109.7	N1—C1—H1A	109.6
N1—C2—H2B	109.7	C4—C1—H1A	109.6
C3—C2—H2B	109.7	N1—C1—H1B	109.6
H2A—C2—H2B	108.2	C4—C1—H1B	109.6
N2—C4—C1	110.25 (15)	H1A—C1—H1B	108.1
N2—C4—H4A	109.6	C8—C7—C6	120.75 (19)
C1—C4—H4A	109.6	C8—C7—H7	119.6
N2—C4—H4B	109.6	C6—C7—H7	119.6
C1—C4—H4B	109.6	C10—C9—C8	120.9 (2)
H4A—C4—H4B	108.1	C10—C9—H9	119.5
N4—C14—N4	0.00 (17)	C8—C9—H9	119.5
N4—C14—C13	128.81 (17)	C15—C16—H16A	109.5
N4—C14—C13	128.81 (17)	C15—C16—H16B	109.5
N4—C14—Cl1	111.18 (13)	H16A—C16—H16B	109.5
N4—C14—Cl1	111.18 (13)	C15—C16—H16C	109.5
C13—C14—Cl1	120.01 (14)	H16A—C16—H16C	109.5
O2—C12—N3	119.11 (17)	H16B—C16—H16C	109.5
O2—C12—C13	124.56 (17)	C15—C17—H17A	109.5
N3—C12—C13	116.32 (16)	C15—C17—H17B	109.5
C7—C6—C5	120.48 (19)	H17A—C17—H17B	109.5
C7—C6—H6	119.8	C15—C17—H17C	109.5
C5—C6—H6	119.8	H17A—C17—H17C	109.5
C14—C13—C12	114.11 (16)	H17B—C17—H17C	109.5
C14—C13—C15	128.36 (17)		
			
N4—N4—C11—O1	0.0 (5)	N3—C12—C13—C14	4.0 (2)
C14—N4—C11—O1	−176.64 (16)	O2—C12—C13—C15	4.8 (3)
C14—N4—C11—N4	0 (100)	N3—C12—C13—C15	−174.41 (15)
N4—N4—C11—N3	0.0 (4)	C4—N2—C3—C2	55.49 (19)
C14—N4—C11—N3	2.5 (2)	N1—C2—C3—N2	−58.11 (19)
C12—N3—C11—O1	179.96 (16)	C14—C13—C15—C16	65.7 (3)
C12—N3—C11—N4	0.8 (3)	C12—C13—C15—C16	−116.20 (19)
C12—N3—C11—N4	0.8 (3)	C14—C13—C15—C17	−65.7 (3)
C5—N1—C2—C3	−165.61 (15)	C12—C13—C15—C17	112.4 (2)
C1—N1—C2—C3	60.88 (19)	C9—C10—C5—C6	1.1 (3)
C3—N2—C4—C1	−54.5 (2)	C9—C10—C5—N1	−176.58 (17)
C11—N4—C14—N4	0 (100)	C7—C6—C5—C10	−1.1 (3)
N4—N4—C14—C13	0.0 (3)	C7—C6—C5—N1	176.42 (17)
C11—N4—C14—C13	−2.5 (3)	C2—N1—C5—C10	164.11 (16)
N4—N4—C14—Cl1	0.0 (3)	C1—N1—C5—C10	−64.4 (2)
C11—N4—C14—Cl1	177.09 (13)	C2—N1—C5—C6	−13.4 (2)
C11—N3—C12—O2	176.56 (16)	C1—N1—C5—C6	118.11 (19)
C11—N3—C12—C13	−4.2 (3)	C5—N1—C1—C4	165.63 (15)
N4—C14—C13—C12	−0.9 (3)	C2—N1—C1—C4	−59.98 (19)
N4—C14—C13—C12	−0.9 (3)	N2—C4—C1—N1	56.0 (2)
Cl1—C14—C13—C12	179.55 (12)	C9—C8—C7—C6	0.4 (3)
N4—C14—C13—C15	177.27 (17)	C5—C6—C7—C8	0.4 (3)
N4—C14—C13—C15	177.27 (17)	C5—C10—C9—C8	−0.3 (3)
Cl1—C14—C13—C15	−2.3 (3)	C7—C8—C9—C10	−0.5 (3)
O2—C12—C13—C14	−176.82 (17)		

### (II) 4-Phenylpiperazin-1-ium 6-chloro-5-isopropyl-2,4-dioxopyrimidin-1-ide Hydrogen-bond geometry (Å, º)

**Table:**

*D*—H···*A*	*D*—H	H···*A*	*D*···*A*	*D*—H···*A*
N2—H2*N*···N4	0.89	1.93	2.813 (2)	174
N2—H3*N*···O1^i^	0.89	1.84	2.705 (2)	164
N3—H3···O2^ii^	0.86	1.98	2.834 (2)	174
C3—H3*A*···O2^iii^	0.97	2.54	3.394 (2)	147

Symmetry codes: (i) −*x*+1, *y*−1/2, −*z*+1/2; (ii) −*x*+1/2, −*y*+5/2, −*z*+1/2; (iii) *x*+1/2, −*y*+2, *z*.
